# The Mediating Role of Responsible Innovation in the Relationship between Stakeholder Pressure and Corporate Sustainability Performance in Times of Crisis: Evidence from Selected Regions in China

**DOI:** 10.3390/ijerph18147277

**Published:** 2021-07-07

**Authors:** Hong Tian, Jiahui Tian

**Affiliations:** Business School, Jilin University, Changchun 130012, China; tianhong2919@163.com

**Keywords:** responsible innovation, stakeholder pressure, corporate sustainability performance, flexible routine replication, corporate social responsibility

## Abstract

Responsible innovation, as a new management paradigm that balances the need for profit growth and the appeal of social value, plays an important role in taking into account corporate economic, social and environmental performance. It provides new ideas for driving enterprises to become more risk-resistant and sustainable in times of crisis. However, existing research on responsible innovation has mostly focused on content issues, and there is a lack of sufficient research and empirical studies on its effectiveness in business organizations. Based on the stakeholder theory and the research logic of “pressure–behavior-performance”, this study investigates the formation mechanism of responsible innovation and its impact on corporate performance. Through empirical research on 306 Chinese sample data, the results show that stakeholder pressure has a positive impact on corporate sustainability performance and responsible innovation plays a partially mediating role in this relationship. Flexible routine replication positively moderates the relationship between stakeholder pressure and responsible innovation, while positively moderating the mediating role that responsible innovation plays between stakeholder pressure and corporate sustainability performance. This study contributes to helping enterprises recognize the importance of responsible innovation in responding to stakeholder pressure and promoting corporate sustainability performance in times of crisis.

## 1. Introduction

The spread of COVID-19 has caused dramatic dynamic environmental changes, changeable market demand and systematic economic stagnation, which have brought great challenges to the survival and development of science and technology enterprises. In times of crisis, how to enhance the ability of enterprises to resist risks while maintaining sustainable production and development has become one of the focuses of research. Innovation is an effective way for enterprises to cope with uncertainty, enabling enterprises to remain dynamic and sustainable [1]. However, the reality is that innovative products such as big data not only bring huge market space and broad development prospects, but also come with a scientific and ethical crisis that challenges the legal and moral bottom line, such as information security and privacy leakage. With the increasing importance of stakeholders to corporate social responsibility and the continuous emergence of ethical dilemmas, academic and business scholars begin to pay attention to the “distinction between justice and benefit” in the new market environment, and to rethink the fundamental issue of “interest and morality”. How can we avoid the “Collingridge’s dilemma” [2] while enjoying the dividends of innovation? How to effectively address the reasonable balance between current innovative development and visionary planning, so as to enhance the ability of enterprises to resist risks? How to take into account the harmonious coexistence of economic, social and environmental benefits, so as to achieve sustainable development? These issues have become important topics that need to be addressed urgently.

The European Commission’s Horizon 2020 Framework Plan proposes the concept of “Responsible Innovation” (RI), which suggests that there should be cooperation and matching between society and science and technology [3,4]. Responsible innovation emphasizes meeting future needs through collective management, and the process and result of innovation should be morally acceptable, sustainable and socially desirable [5]. Given the “balance” thought contained in responsible innovation, this concept has been introduced in business management research as a new management paradigm [6,7]. Responsible innovation will mitigate or even reverse the negative externalities of corporate activities and will create both economic benefits and business opportunities for enterprises [8,9]. However, much of the research on responsible innovation is currently focused on science and technology innovation [10] rather than business-driven innovation [11]. Enterprises are an important source of innovation [8], but for enterprises that pay attention to efficiency and cost, whether and why they are willing to adopt responsible innovation activities is an issue to be discussed. In addition, researchers have focused on the evaluation criteria and content issues of responsible innovation [12,13], but pay little attention to its implementation path and mechanism, that is, there is a lack of research on the motivation and impact of responsible innovation in the business field.

Stakeholder theory provides a theoretical basis for integrating business and social domain research [14]. Stakeholders provide resources and support for the long-term sustainability of enterprises and at the same time require enterprises to actively respond to the value proposition of the stakeholders [15]. Given that stakeholder rewards and penalties for sustainable performance will threaten the survival and growth of enterprises, enterprises are increasingly focusing on sustainability activities and devoting more resources to developing strategies, policies and practices that are consistent with sustainability and social responsibility goals in response to stakeholder pressure [16,17]. As a deliberative innovation mode in which stakeholders are included in innovation activities, responsible innovation can meet both economic benefits and sustainable development goals [7], and its innovation process and results will be affected by the value demands of stakeholders. It can be seen that there is an obvious logical relationship between stakeholder pressure and responsible innovation. Therefore, this study considers the role of stakeholder pressure on responsible innovation from a stakeholder perspective and then explores the impact of adopting this innovation model on the sustainable performance of enterprises. Furthermore, the ability of enterprises to effectively organize, search and reconstruct knowledge to match and meet the needs of responsible innovation also have a significant impact on corporate innovation effectiveness and sustainability performance [18]. Flexible routine replication, which relies on exploration capability, emphasizes more on the dynamic capability of enterprises, and can help enterprises break the old knowledge coupling, generate new knowledge combinations and create value [19]. Therefore, flexible routine replication is a new means for enterprises to create and solve problems [20], which is helpful for enterprises to acquire diversified knowledge to solve the problem of value co-creation between stakeholders and enterprises. This study also explores the moderating role of flexible routine replication in these influence paths.

In conclusion, based on the stakeholder theory, this study introduces responsible innovation into the research field of corporate strategic management, focuses on the promoting effect of stakeholder pressure on responsible innovation and the impact of responsible innovation on the sustainable performance of enterprises, and discusses the influencing mechanism under the contingency effect of flexible routine replication. This study can make contributions in the following aspects: First, it provides a new perspective on the “righteousness and profit debate”, which is helpful for enterprises to solve the problem of harmonious coexistence of interests and morality. Second, it expands the cross research between stakeholder theory and corporate innovation management, which helps to promote the multi-subject co-governance of corporate innovation. Third, the knowledge and capability factors are extended to the research framework of responsible innovation, and the importance of knowledge management is revealed. In addition, this study provides a practical insight for balancing innovation independence and social responsibility, and promotes corporate sustainable development in times of crisis.

## 2. Theory and Hypotheses

### 2.1. Responsible Innovation

The concept of responsible innovation, proposed by Stilgoe et al. [5], has been widely accepted by academics to refer to the collective management of current science and innovation to meet future needs, and is more broadly defined as “innovation with society and innovation for society”. Responsible innovation is a new concept based on governance approaches and innovation assessment, democratizing innovation through deliberative forms of governance such as stakeholder and public participation, specifically aiming to incorporate ethical and social concerns at the outset of innovation in order to avoid negative impacts of innovation [21,22]. Lubberink et al. [7] further propose a framework for responsible innovation in a business context and provide practices conducive to the implementation of responsible innovation. In the business context, responsible innovation covers the whole process of enterprise innovation, including stakeholder participation and prediction at the beginning of innovation, reflection and introspection in the innovation process, and the conformity of innovation results with moral and social expectations [9,12,23].

Unlike previous “risk management frameworks” that focused only on avoiding harm [24], or “social innovation” and “shared value” studies that focused only on doing good [25], the governance and responsibility logic contained in responsible innovation can simultaneously promote the dual results of avoiding harm and doing good [7,8,26]. Compared with the traditional innovation model, the characteristics of responsible innovation are mainly reflected in four aspects: anticipation, reflexivity, inclusion and responsiveness [5]. Anticipation refers to the systematic thinking of any possible impact of innovation in order to predict potential problems of innovation and evaluate available alternatives, aiming to form “proactive governance” [7]. Reflexivity implies that enterprises confront their activities, commitments and assumptions and become aware of the limitations of their knowledge. In addition, enterprises should examine how their value systems and beliefs influence the development of corporate innovation, not only by fulfilling their role tasks but also by taking on broader ethical responsibilities [5,21]. Inclusion implies that enterprises should leverage multi-stakeholder relationships to include lay members in responsible innovation activities, seeking to diversify inputs and delivery to governance [10]. Responsiveness refers to the fact that responsible innovation requires the ability to change shape or direction in response to stakeholder and public values and changing circumstances [27].

Studies have focused on the theoretical framework of responsible innovation [7], the innovation process [10], governance mechanisms, and from institutional scenarios, resource capacity [6,23] and corporate social responsibility [28] perspectives to explore the formation mechanism of corporate responsible innovation [29]. Based on the stakeholder theory, this study presents responsible innovation as a way for enterprises to respond to stakeholder demands and to balance the needs of corporate development with social value expectations to achieve strategic goals for sustainable development.

### 2.2. Stakeholder Pressure and Corporate Sustainability Performance

Corporate sustainability focuses on the integration of economic prosperity, environmental protection and social progress [17,30], which is considered as a corporate strategy for enterprises to seek the best business practices to meet and balance the needs of current and future stakeholders [31,32,33]. Corporate sustainability performance measures the extent to which enterprises include economic, environmental, social and governance factors in their operations [34,35], as well as the final impact these factors have on the enterprises and society, including economic performance, social performance and environmental performance [32,36,37]. Horisch et al. [38] have shown that the central themes of stakeholder theory and corporate sustainability research are similar in terms of creating long-term environmental, social and economic value for stakeholders and enterprises. Stakeholders are institutions, organizations, communities and individuals that may influence or be affected by specific organizations. Stakeholder pressure can prompt enterprises to adopt strategies and practices in line with the goal of social responsibility, to reduce agency problems while safeguarding their interests [15,39].

Currently, corporate responses to sustainability issues are influenced by the sustainability concerns of a growing number of stakeholders, and responsible behavior has been recognized as essential for corporate success and survival [9,40,41]. Enterprises must clarify their important stakeholder relationships, systematically evaluate the impact of corporate actions on stakeholders, and then actively adjust their responsible behaviors to maintain a mutually beneficial strategic partnership with stakeholders or reconcile differences [12,42]. On the one hand, the action of enterprises responding to the demands of stakeholders helps enterprises mobilize and share the capital, knowledge and technology of stakeholders [43], which helps enterprises form strategic partnerships with stakeholders [10,14] while promoting the formation of specific environmental and social capabilities [10,14]. In this way, enterprises can reduce the cost of solving sustainable development problems and improve their sustainable performance [8]. Moreover, enterprises focusing on growth and development integrate stakeholder pressure with enterprises’ value chain by implementing social responsibility activities related to their main business [44], that is, creating shared value through strategic corporate social responsibility [25,45,46,47]. This multi-objective strategic model cannot only focus on and respond to the changing social and environmental concerns of stakeholders, but also provide opportunities and benefits for the core business of enterprises [48]. On the other hand, stakeholder pressure helps enterprises understand the preferences of stakeholders such as governments and consumers in terms of sustainability [49]. To enhance the effectiveness of corporate sustainability strategies and respond to stakeholders’ sustainability needs, threats and opportunities to corporate sustainability strategies must be identified, as well as reducing and avoiding corporate risks and uncertainties [18,41]. Furthermore, enterprises’ initiatives that incorporate social responsibility and stakeholder pressure into strategic corporate thinking can be seen as doing the right thing, thus helping to protect corporate reputation, improve corporate image and ensure the long-term sustainability of the company’s objectives [9,23]. In summary, stakeholder pressure motivates enterprises to respond and fulfill their social and environmental responsibilities while focusing on economic efficiency, which helps enterprises improve corporate sustainability performance. Therefore, this paper proposes the following hypothesis:

**Hypothesis** **1 (H1).** *Stakeholder pressure is positively related to corporate sustainability performance*.

### 2.3. The Mediating Effect of Responsible Innovation in an Association between Stakeholder Pressure and Corporate Sustainability Performance

According to the stakeholder theory, organizations establish relationships with several stakeholder groups and stakeholders can influence organizations’ decisions [15]. On the one hand, as a form of governance in which enterprises include stakeholders and the public in a deliberative innovation model, responsible innovation can respond to the demands of stakeholders by discussing with stakeholders the underlying norms and values that can guide innovation in the desired direction [7]. Enterprises integrate the sustainability goals of different stakeholders, thus prompting corporate innovation processes and outcomes that are consistent with stakeholders’ expectations of social and environmental responsibility [6,50,51]. On the other hand, responsible innovation facilitates the exchange of resources and capabilities between enterprises and stakeholders. Enterprises reflect on potential problems in the existing innovation model, while assessing the feasibility of alternative solutions, thus achieving the goal of improving the efficiency and effectiveness of corporate innovation. In addition, responsible innovation can enhance investors’ understanding and recognition of enterprise innovation by reducing the information asymmetry and uncertainty of innovation, so as to improve enterprise performance [36]. In other words, responsible innovation conforms to the “triple bottom line” principle: taking sustainable development as the ideal result of innovation, promoting the integration of responsibility ethics and core business, and innovating achievements that cover three dimensions—economy, environment and society [7,9,26,52].

Specifically, enterprises include different stakeholders and the public at the outset of innovation activities to establish a forward-looking model of responsibility, thus increasing the likelihood of anticipating and discerning how innovation can benefit society and prevent any negative consequences from occurring [21,53]. Stakeholder pressure can promote better learning and decision-making of enterprises [54], which not only encourages enterprises to critically examine the social, environmental, political and moral impacts brought by innovation [55], but also urges enterprises to rethink the relationship between innovation and social needs [7]. Enterprises take actions and adjust according to new knowledge, new ideas, new perceptions and new norms emerging in the innovation process, so as to make innovation adapt to changes and meet new demands [56], and ensure the ethical appropriateness of enterprise products, and the acceptability of relevant performance and quality [9,57,58]. This process not only helps enterprises to create benefits, but also helps enterprises to organize resources and guide innovation in the direction of protecting the natural environment and increasing social welfare as quickly as possible [5,59].

In summary, this study believes that enterprises establish a continuous, transparent and interactive innovation process consistent with social values, needs and expectations through responsible innovation [12]. The process can help enterprises anticipate and respond to ethical, social and environmental concerns, thus not only responding to the pressure of stakeholder expectations but also balancing economic efficiency gains with the need for social values. This measure takes into account the needs of enterprises’ economic development and social ethical responsibilities, and will create environmental and social performance while improving the efficiency and benefits of enterprises’ innovation, that is, improve corporate sustainability performance. Therefore, this paper proposes the following hypothesis:

**Hypothesis** **2 (H2).** *Stakeholder pressure influences corporate sustainability performance through the mediating role of responsible innovation*.

### 2.4. The Moderating Effect of Flexible Routine Replication

Organizational routine is repetitive, identifiable and interdependent patterns of behavior involving multiple actors [60,61], and are divided into two categories based on a capability perspective: conventional routine and flexible routine [62]. Conventional routine refers to the general competence patterns of organizations, which contain stable, explicit, continuous and solidified knowledge, while flexible routine emphasizes the dynamic capabilities of enterprises, which contain more complex, implicit and diverse knowledge [20]. In an unstable environment, flexible routines not only help to adjust the original knowledge templates but also promote exploratory learning and searching for heterogeneous knowledge to optimize the allocation of resources and expand organizational boundaries [63]. In this situation, flexible routines will exhibit a high level of replication [20,64]. Flexible routine replication is a new means for enterprises to create problem-solving that can help them break old knowledge coupling, generate new knowledge combinations and create value [19].

Responsible innovation focuses on the interaction between innovation structures and stakeholders, which requires enterprises to be able to respond quickly to new ideas, knowledge and norms emerging in the interaction, and the ever-changing external environment, to make responsible decisions that allow innovation to adapt to change and meet new needs [7,12]. On the one hand, enterprises with a high degree of flexible routine replication can quickly respond to dynamic changes in the environment, capture complex and changing knowledge needs, and then conduct exploratory organizational learning and extensive knowledge search [65]. On the other hand, enterprises promote knowledge transfer and knowledge creation through the splitting and reorganization of existing knowledge modules and the reallocation of organizational resources [66] to improve the efficiency of enterprise innovation model transformation [67]. The development and deployment of flexible routines help improve the firm’s capacity for technological change and flexible adaptation. Such dynamic capabilities provide the knowledge, resources and foundation to respond quickly to changing needs, facilitate the inclusion of stakeholders in a deliberative innovation model form of governance and address the opportunities and challenges presented by stakeholder pressure. In addition, enterprises with a high degree of flexible routines replication are more likely to develop a flexible, risk-taking, innovative organizational climate and learning culture. When facing the demands of stakeholders, this type of enterprise is more innovative and proactive and tends to take actions to meet the challenges. Therefore, enterprises with a high degree of flexible routine replication are more inclined to adopt a responsible innovation approach to solve the dilemma of economic benefit, social prosperity and environmental protection. Therefore, this paper proposes the following hypothesis:

**Hypothesis** **3 (H3).** *Flexible routine replication would positively moderate the relationship between stakeholder pressure and responsible innovation*.

Based on the above assumptions of mediating and moderating effects, this study further proposes a moderated mediating effect model. Corporate sustainability means that enterprises must place sustainability goals at the core, rather than enhancing their success “through additional social and environmental goals as a form of responsive corporate social responsibility” [68]. This requires enterprises to have the ability and knowledge to change their existing innovation mode, and at the same time, pioneering independent attempts and efforts are needed to adapt to the special and constantly changing external environment [69]. Enterprises with a high degree of flexible routine replication have low obstacles and resistance to the acquisition, generation, integration and replacement of new knowledge, which enables enterprises to have strong adaptability and fast organizational knowledge update, and focus on the long-term development of enterprises [66]. This means that enterprises have the ability to acquire heterogeneous knowledge through exploratory learning, and adjust their innovation model by integrating existing resource allocation and knowledge reserve, so as to provide an execution basis for meeting the pressure from stakeholders to balance economic, environmental and social performance. In summary, enterprises with high levels of flexible routine replication are more likely to catalyze responsible innovation when faced with stakeholder pressure, which in turn enhances corporate sustainability performance. Therefore, this paper proposes the following hypothesis:

**Hypothesis** **4 (H4).** *Flexible routine replication would positively moderate the mediating role of responsible innovation between stakeholder pressure and corporate sustainability performance*.

**Figure 1:**
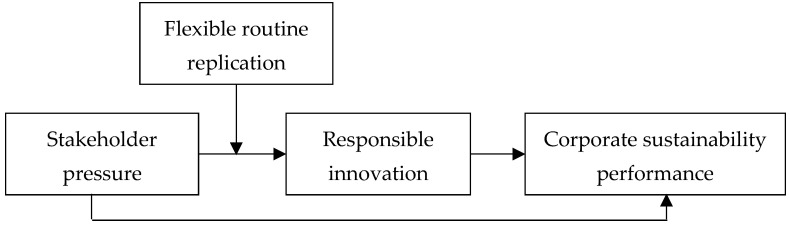
Theoretical model.

The theoretical model diagram of this study is shown in Figure 1.

## 3. Methodology

### 3.1. Sample Selection and Data Collection

This study covers technology-based enterprises in the Yangtze River Delta region, the Pearl River Delta region and the Northeast region of China. The Yangtze River Delta region and the Pearl River Delta region have high innovation activity and strong R&D foundations [70,71]. These two regions are the main gathering places of science and technology enterprises. The Northeast region of China used to be famous as an old industrial base in China, with strong scientific and technological strength and profound industrial deposits. There are also numerous technology-based enterprises. Therefore, these three representative regions were selected as data sources in this study. Influenced by COVID-19, online and offline data collection methods were adopted in this study. To ensure that the research respondents have a clear and comprehensive understanding and knowledge of their companies, the study invited the chairman, president and managers in charge of R&D to fill in the answers based on the actual situation of their companies in the past three years. In China, the COVID-19 epidemic started in late 2019 and lasted for nearly a year and a half by the time the questionnaire was sent out. The three-year data range of 2019, 2020 and 2021 investigated in this study can adequately cover the whole process from the emergence of the epidemic to the outbreak and then to the long-term existence of the epidemic. Before the start of the research, the project team clarified the research intention, purpose, confidentiality measures and precautions to the respondents, emphasizing that the research was conducted anonymously and the questionnaire was only used for scientific research and no personal or corporate information would be disclosed. The questionnaire distribution of this study began in early March 2021 and ended in late April 2021. A total of 600 questionnaires were distributed in this study, and after deleting questionnaires with incomplete or regular responses, 306 valid questionnaires were recovered, with a valid recovery rate of 51.0%. The results of the descriptive statistics of the sample are as follows: in terms of enterprise age, 39.54% are 5 years or less, 42.81% are 6–10 years, and 17.65% are 11 years or more. In terms of enterprise size: 39.21% for 100 persons and below, 33.01% for 101–300 persons, and 27.78% for more than 300 persons. Enterprise nature: state-owned/collective accounted for 24.51%, private 57.84%, foreign/joint venture 17.65%.

### 3.2. Measures

To ensure the reliability and validity of the questionnaire, well-established scales published in international mainstream academic journals were selected for the measurement of each variable. In this study, a translation team including doctoral students majoring in business management and English was established to form a Chinese questionnaire according to the standard translation–back translation procedure. Before the questionnaire was distributed, the study asked professors in business management to review the questionnaire to ensure its relevance in the Chinese context. The questionnaire was scored on a Likert7 scale, from 1 to 7, indicating “strongly disagree” to “strongly agree”.

Depending on the degree of influence on the firm, stakeholders are divided into primary stakeholders (including shareholders, investors, employees, customers and government), which are critical to the firm’s survival, and secondary stakeholders (media and nonprofit organizations), which can influence public opinion and thus damage or enhance the firm’s reputation [6]. Given the greater influence of primary stakeholders on corporate strategies and decisions in the Chinese context, this study focused on the role of primary stakeholder pressure. Helmig et al.’s [6] 10-item scale for measuring key stakeholder pressure was selected for this study, with representative items such as “Our customers’ purchasing habits are changing to support responsible corporations (e.g., fair trade coffee)”. Responsible innovation is based on the four-item scale selected by Stilgoe et al. [5] and Cao et al. [23] in which the representative questions include “In the early stage of innovation activities, companies can conduct a forward-looking analysis of the future impact of innovation activities, so as to guide them in an ethically acceptable and socially satisfactory direction, and achieve controlled risks in innovation activities”. Flexible routine replication is based on the study of Wei and Dang [65] and a four-item scale was selected, which is representative of questions such as “Firms are able to quickly promote and apply new organizational norms to meet internal and external challenges”. Corporate sustainability performance is based on Li et al.’s [36] study, an 11-item scale was selected, including 4 items for environmental performance, 4 items for economic performance, and 3 items for social performance, with representative items such as “Our company has reduced consumption of hazardous and toxic substances”, “Our company develops community economic activities and provides more employment opportunities”. The control variables were selected as firm age, firm size and firm nature. Among them, the age of the enterprise is expressed by the number of years of establishment, the size of the enterprise is measured by the number of people in the enterprise, and the nature of the enterprise is divided into state-owned enterprises, foreign-funded enterprises, joint ventures and private enterprises.

## 4. Results and Analysis

### 4.1. Reliability and Validity

This study used SPSS 23.0 to conduct reliability and validity tests. The results are shown in Table 1. The Cronbach’s α coefficients of stakeholder pressure, responsible innovation, flexible routine replication and corporate sustainability performance were all greater than 0.7. The composite reliability of each construct was greater than the recommended level of 0.7, indicating that the questionnaire had good reliability. In terms of validity, this study used the double-blind translation method and appropriately adjusted and modified the relevant questions according to the Chinese scenario, with good content validity. AVE was all greater than the recommended 0.5 level, indicating good convergent validity.

### 4.2. Analysis and Results

Table 2 shows the means, standard deviations and correlation matrix of the variables. Stakeholder pressure was significantly correlated with responsible innovation (r = 0.593, *p* < 0.01), stakeholder pressure was significantly correlated with corporate sustainability performance (r = 0.608, *p* < 0.01) and responsible innovation was significantly correlated with corporate sustainability performance (r = 0.540, *p* < 0.01). These results initially validate the hypotheses of this study, which were further tested by using regression analysis. Furthermore, from the AVE values in Table 1, it can be concluded that the square root of AVE for stakeholder pressure, responsible innovation, flexible routine replication and corporate sustainability performance are 0.731, 0.784, 0.793 and 0.713. When the AVE square root value of the factor is greater than the correlation value between the factor and other factors, it can indicate that it has good discriminative validity. Therefore, the discriminant validity of the variables in this study is good.

### 4.3. Hypotheses Testing

In this study, multiple linear regression methods are used to test each hypothesis, and the results are presented in Table 3. This study first centralizes the variables used and constructs the interaction term of stakeholder pressure and flexible routine replication after centralization to reduce the multi-collinearity between the variables. M1 is the effect of control variables on responsible innovation, M2 is the effect of independent variables on responsible innovation and M4 is the effect of the interaction term of stakeholder pressure and flexible routine replication on responsible innovation. M5 is the effect of control variables on corporate sustainability performance, M6 is the effect of independent variables on corporate sustainability performance and M7 is the effect of independent and mediating variables on corporate sustainability performance. All models are significant except for models M1 and M5.

In M6, stakeholder pressure has a significant positive effect on corporate sustainability performance (β = 0.605, *p* < 0.001), and hypothesis H1 is supported. M5 shows that firm nature has a significant impact on corporate sustainability performance. Therefore, in this study, further tests were conducted by grouping according to firm nature. The total number of samples is 306. After grouping the enterprises according to their nature, the sample size is 75 for state-owned/collective enterprises, 177 for private enterprises, and only 54 for foreign-funded/joint ventures. First, the LSD pairwise t-test was used for comparison, and the results are shown in Table 4. Through data comparison, it is found that the corporate sustainability performance of state-owned/collective enterprises is more outstanding than that of private enterprises. Next, the main hypothesis test was conducted according to enterprises with different natures. The results are shown in Table 5. The corporate sustainability performance of state-owned/collective enterprises and private enterprises are affected by stakeholder pressure, and the regression coefficient of state-owned/collective enterprises is higher. This finding is consistent with the current development in China that state-owned/collective enterprises will take on more corporate social responsibility and respond to stakeholder demands than private enterprises, resulting in a more significant improvement in corporate sustainability performance. The reason for the insignificance of foreign/joint ventures may be due to the small sample size as the ratio of the number of foreign/joint ventures to the number of variables after grouping does not reach 15:1 [72].

M2 in Table 3 shows that stakeholder pressure is a good predictor of responsible innovation (β = 0.590, *p* < 0.001). M7 builds on M6 by including responsible innovation in the regression equation, and responsible innovation has a positive effect on corporate sustainability performance (β = 0.277, *p* < 0.001), and the effect of stakeholder pressure on corporate sustainability performance remains significant but decreases (β = 0.441, *p* < 0.001), assuming that H2 is supported.

This study further examined the mediating effect by Bootstrap method [73,74], and the total effect of stakeholder pressure on corporate sustainability performance was 0.605, the direct effect was 0.442 and the indirect effect was 0.163 (95% confidence interval is [0.059, 0.293]), indicating that responsible innovation plays a partial mediating role.

**Figure 2:**
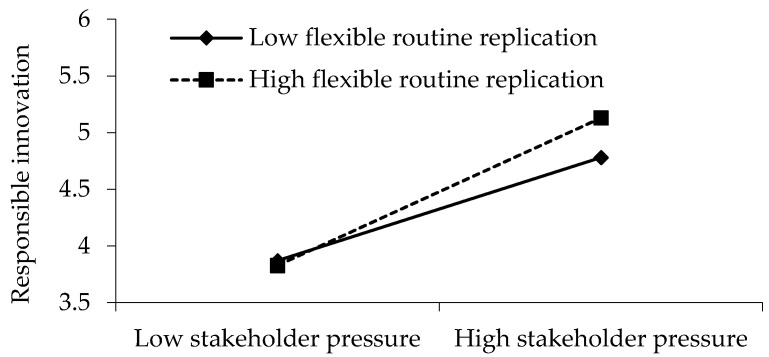
Diagram of regulating role.

M4 includes the interaction term between stakeholder pressure and flexible routine replication in the regression equation with responsible innovation as the dependent variable, and the interaction term is significant (β = 0.098, *p* < 0.05), indicating that flexible routine replication plays a moderating role between stakeholder pressure and responsible innovation. This study further verified the moderating effect by dividing the data into two groups, high and low, by adding and subtracting one standard deviation from the mean value of flexible routine replication, which is shown in Figure 2. The results show that the higher the level of flexible routine replication, the stronger the effect of stakeholder pressure on responsible innovation. Therefore, flexible routine replication plays a positive moderating role between stakeholder pressure and responsible innovation. Therefore, H3 is supported.

This study used the PROCESS procedure to test the mediated moderation model. The results are shown in Table 6. When responsible innovation is the mediator, flexible routine replication moderates the indirect effect of stakeholder pressure on corporate sustainability performance with an INDEX of 0.034 (95% CI= [0.006, 0.198]). Specifically, the moderating effect of flexible routine replication on the indirect effect is insignificant when flexible practice replication takes the mean minus one standard deviation (95% CI= [−0.007, 0.215]), while the moderating effect of flexible practice replication on the indirect effect is significant when flexible practice replication takes the mean plus one standard deviation (95% CI= [0.067, 0.431]); therefore, H4 is supported.

## 5. Discussion

This study explores the important role of responsible innovation in dealing with the challenges of stakeholder pressure, and how to transform stakeholder pressure into a driving force for corporate sustainability performance. Based on stakeholder theory, this study constructs a logical framework of “stakeholder pressure–responsible innovation– corporate sustainability performance”. Under the boundary condition of flexible routine replication, we investigate the impact of stakeholder pressure on responsible innovation and its effect on corporate sustainability performance. The main finding is that stakeholder pressure has a positive impact on corporate sustainability performance and that responsible innovation plays a partially mediating role in the relationship. Flexible routine replication not only positively moderates the relationship between stakeholder pressure and responsible innovation, but also mediates the role of responsible innovation in mediating the relationship between stakeholder pressure and corporate sustainability performance.

This study explores the effectiveness of responsible innovation in balancing interests and ethics. On the one hand, the important role of responsible innovation in dealing with the challenge of stakeholder pressure and transforming the pressure into the driving force of sustainable development is revealed and empirically tested in this study. When an enterprise actively responds to the demands of stakeholders and takes responsible innovation, this action helps the enterprise find new development opportunities in the challenges, and promote the formation of a mutually beneficial and win–win strategic partnership with stakeholders, so as to achieve value co-creation and improve the corporate sustainability performance. This study responds to the call of Lubberink et al. to study responsible innovation in the business context [7]. In addition, although some scholars have paid attention to the close relationship between stakeholders and responsible innovation (e.g., [10,12]), this study further composes and empirically tests the relationship between the two.

On the other hand, this study regards responsible innovation as a kind of corporate management paradigm, which is an active and long-term strategic choice of enterprises [12], rather than a response under critical circumstances. The content of this study shows that even if the epidemic crisis has a profound impact on the business environment [75], enterprises’ actions of actively responding to the pressure from stakeholders and taking responsible innovation will still show effectiveness. This study echoes and extends He and Harris’ observation that “the COVID-19 pandemic offers a great opportunity for companies to contribute to addressing global social and environmental challenges” [76]. Responsible innovation can not only help enterprises promote social prosperity and environmental protection, but also guarantees the survival and development of enterprises, and effectively improves the ability of enterprises to resist risks in critical situations.

### 5.1. Theory Contributions

First, responsible innovation is introduced into the field of corporate strategic management research as a new innovation management paradigm for enterprises to solve the problem of harmonious coexistence of profit and ethics and promote sustainable development. The existing responsible innovation mainly focuses on the policy level, and the theoretical research lags behind and mainly adopts qualitative methods (e.g., [8,10,68]). Few scholars have discussed the applicability and effectiveness of responsible innovation in the context of Chinese organizations (e.g., [23]). This study quantitatively clarifies the positive role of responsible innovation in responding to stakeholder demands, balancing economic, environmental and social values, and enhancing sustainable corporate performance in China. The findings of this study provide a reference for promoting deeper theoretical research on responsible innovation and helps guide enterprises to properly handle the relationship with stakeholders, inspire value co-creation between enterprises and stakeholders, and turn pressure into motivation to improve sustainable performance [8,52].

Second, it expands the intersection study of stakeholder theory and corporate innovation management. The atomic innovation governance of a single subject has revealed its limitations in the new economic development, and innovation governance urgently needs to be transformed to innovation co-governance with the joint participation of multiple subjects. Different from social innovation [77], green innovation and other innovation modes [78] that focus on specific stakeholders and solve specific problems, responsible innovation can broadly absorb the value demands of multi-stakeholders and make it possible for multi-stakeholders to co-govern [7]. Therefore, this study provides a general governance framework for enterprises to balance responsibility and innovation through the study of responsible innovation, which helps to promote the integration of stakeholder pressure and corporate innovation management and strengthen the continuous optimization and multi-subject co-governance of the whole innovation process.

Third, the factors of knowledge and ability are extended to the research framework of corporate social responsibility and business ethics that provide a new theoretical perspective for the research in this field. Existing studies mostly focus on business ethics from the perspectives of corporate values, culture and leadership style, etc. (e.g., [79,80,81]), while this study focuses on competency factors. By combining capability factors with responsible innovation, this study clarifies the catalytic role of flexible routine replication in the path of responsible innovation, and makes clear that dynamic capabilities and knowledge resources are one of the operational foundations for enterprises to successfully respond to new ideas, new norms and rapidly changing external environments. The conclusions of this study provide a new way of thinking for improving the effectiveness of responsible innovation and corporate social responsibility strategies, and solving the problem of decoupling-related policy formulation and implementation results of business ethics.

### 5.2. Practice Contributions

First, it provides ideas for solving the dilemma between enterprise benefit growth and social responsibility fulfillment. In recent years, China’s industrial structure has shifted from a stage of rapid growth to a stage of high-quality development. At the same time, the government has emphasized the development of a circular economy and an environment-friendly economic model. However, environmental pollution problems still persist. At the same time, some enterprises take responsible behavior to gain legitimacy, but it brings a huge cost to enterprises. As an innovative behavior pattern that can not only produce economic benefits but also sustainable social values, responsible innovation can effectively solve the problem of “the difference between justice and benefit” and the disconnection between “responsibility” and “innovation”, and guide enterprise innovation to develop in a morally acceptable, socially desirable and sustainable direction. Therefore, governments and enterprises in China and other countries can learn from and promote responsible innovation models that embed business ethics into the business process. Just as the Chinese traditional culture mentioned, “Justice to generate profits, profits to enrich the people”, and then achieve high-quality economic development.

Second, a collaborative innovation and governance system should be created with enterprises as the main body and a wide range of stakeholders involved. On the one hand, enterprises should increase communication links with stakeholders interactively and openly, improve the transparency of information related to technological innovation, and reduce the possibility of conflicting value claims arising from information asymmetry. Moreover, enterprises should proactively understand and grasp the needs of stakeholders and promote a cooperative business model to achieve complementary resources, knowledge sharing and value creation between enterprises and stakeholders, thereby jointly creating economic and social value. On the other hand, enterprises should keep track of the dynamic changes of laws, social situations, science and technology and other factors that can significantly affect the enterprise innovation environment in real-time. These measures can help enterprises to keenly capture the problems that will threaten social and environmental sustainability, and to consider and avoid the potential impact of these problems on the development of society and enterprises.

Third, it provides practical inspiration for improving the effectiveness of responsible innovation from the perspective of ability. Adequate knowledge and resources are the basic elements to form responsible innovation, and flexible routine replication is one of the important guarantees for enterprises to organize and manage knowledge flexibly and respond to the pressure of stakeholders. Therefore, enterprises should increase the investment in the knowledge base and innovation management, improve the ability of searching, absorbing and integrating heterogeneous knowledge, and gradually increase the stock of enterprise knowledge. In addition, enterprises should create an open, interactive, transformational organizational atmosphere and an innovative, learning-oriented organizational culture. Enterprise training, intelligence introduction and other measures enhance the divergent innovative thinking and stimulate the subjective initiative of employees. In this way, enterprises are encouraged to break the subjective cognitive limitations, reduce excessive reliance on existing conventions and knowledge, and improve the operability of the implementation of responsible innovation.

### 5.3. Limitations and Future Research

First, the cross-sectional data collection method is adopted in this study, which cannot reflect the long-term and dynamic impact of responsible innovation on corporate sustainability performance. In the future, the tracking research method can be used to explore the continuous impact of responsible innovation on corporate sustainability performance. Second, in terms of sample selection, samples in this study are from Chinese science and technology enterprises, with strong pertinence. For different industries in different emerging economies, the business environment for enterprises is very different. Therefore, future research can be extended to different industries and different countries to further explore the effectiveness of responsible innovation, so as to enhance the universality and practical value of the research. Finally, this study only studies the formation mechanism of responsible innovation from the perspective of stakeholders. In practice, responsible innovation may also be affected by institutional environment, corporate culture and other factors. Therefore, future research needs to combine a variety of theoretical perspectives to deepen the theoretical framework of responsible innovation.

## 6. Conclusions

The focus of this study is on how to solve the problem of “justice and interest debate”, aiming to guide enterprises not only to improve their ability to resist risks but also to promote sustainable development while paying attention to social values and environmental protection. To be specific, this study takes Chinese science and technology enterprises as the research object and discusses the following three issues: First, how to effectively deal with the pressure from stakeholders to facilitate corporate sustainability performance. The second is how responsible innovation balances economic growth and social value creation in order to promote corporate sustainability performance. The third is to discuss when responsible innovation can more effectively promote corporate sustainability performance. This study further promotes the theoretical research of responsible innovation in the business context. In terms of practice, enterprises in China and other countries can get practical inspiration from this paper to reasonably balance interests and ethics and promote high-quality development. It also provides ideas for enterprises to promote corporate sustainability performance in times of crisis.

## Figures and Tables

**Table 1:** Reliability and validity test results.

Variables	Cronbach’s α	AVE	CR	KMO	Variance Interpretation
Stakeholder pressure	0.839	0.535	0.911	0.849	66.763%
Responsible Innovation	0.789	0.614	0.864	0.782	61.372%
Flexible Routine Replication	0.802	0.629	0.871	0.791	62.936%
Corporate sustainability performance	0.915	0.508	0.917	0.906	70.681%

**Table 2:** Descriptive statistical analysis.

	1	2	3	4	5	6	7
1. Firm age	-						
2. Firm size	0.225 **	-					
3. Nature	0.241 **	0.079	-				
4. Stakeholder pressure	−0.018	−0.051	−0.100	-			
5. Responsible innovation	0.045	−0.025	−0.077	0.593 **	-		
6. Flexible routine replication	−0.001	−0.034	−0.086	0.083	0.140 *	-	
7. Corporate sustainability performance	−0.014	0.016	−0.113 *	0.608 **	0.540 **	0.032	-
Mean	1.781	1.886	1.931	4.360	4.275	4.184	4.307
S.D.	0.725	0.812	0.647	0.500	0.742	0.749	0.645

Note: * *p* < 0.05, ** *p* < 0.01 (two-tailed).

**Table 3:** Results of regression analysis.

Variables	Responsible Innovation				Corporate Sustainability Performance		
	M1	M2	M3	M4	M5	M6	M7
Firm age	0.075	0.065	0.063	0.071	0.009	−0.002	−0.020
Firm size	−0.035	−0.007	−0.004	−0.008	0.023	0.051	0.053
Firm nature	−0.092	−0.033	−0.025	−0.019	−0.117 *	−0.056	−0.047
Stakeholder pressure		0.590 ***	0.584 ***	0.553 ***		0.605 ***	0.441 ***
Flexible routine replication			0.090	0.077			
Stakeholder pressure × Flexible routine replication				0.098 *			
Responsible innovation							0.277 ***
R2	0.011	0.355	0.363	0.372	0.013	0.375	0.424
ΔR2	0.002	0.347	0.353	0.359	0.004	0.366	0.414
F	1.157	41.481 ***	34.239 ***	29.477 ***	1.368	45.091 ***	44.183 ***

Note: * *p* < 0.05, *** *p* < 0.001 (two-tailed); The sample size is 306.

**Table 4:** LSD analysis results for different firm natures.

Variables	Firm Nature	Firm Nature	Mean Difference	Sig.
Corporate sustainability performance	state-owned/collective	private	0.222 *	0.012
	state-owned/collective	foreign/joint	0.202	0.078
	private	foreign/joint	−0.020	0.840

Note: * *p* < 0.05.

**Table 5:** The results of grouping main hypothesis test for different firm natures.

Variables	Corporate Sustainability Performance					
	State-Owned/Collective Enterprises		Private Enterprises		Foreign/Joint Venture	
	M8	M9	M10	M11	M12	M13
Firm age	0.016	0.197	−0.043	−0.075	0.077	0.071
Firm size	0.157	0.081	−0.098	0.005	0.260	0.288
Stakeholder pressure		0.697 ***		0.692 ***		−0.122
R2	0.027	0.486	0.012	0.479	0.085	0.099
ΔR2	0.000	0.464	0.001	0.470	0.049	0.045
F	0.997	22.334 ***	1.049	53.012 ***	2.356	1.829
Observations	75	75	177	177	54	54

Note: *** *p* < 0.001 (two-tailed).

**Table 6:** Results of the mediated moderation model.

	Indirect Effects	BootSE	[BootLLCI, BootULCI]
−1 SD	0.119	0.057	[−0.007, 0.215]
0	0.153	0.058	[0.054, 0.281]
+1 SD	0.187	0.092	[0.067, 0.431]

## Data Availability

Data sharing not applicable.
